# Insights from common buzzard broods on the interaction between *Leucocytozoon* infection, watercourse habitats and simuliid blackfly vectors

**DOI:** 10.1016/j.ijppaw.2024.100978

**Published:** 2024-08-24

**Authors:** Anja Wiegmann, Andrea Springer, Meinolf Ottensmann, Tony Rinaud, Oliver Krüger, Christina Strube, Nayden Chakarov

**Affiliations:** a Department of Animal Behaviour, Bielefeld University, Konsequenz 45, 33615, Bielefeld, Germany; b Institute for Parasitology, Centre for Infection Medicine, University of Veterinary Medicine Hannover, Buenteweg 17, 30559, Hannover, Germany; c Joint Institute for Individualisation in a Changing Environment (JICE), University of Münster and Bielefeld University, Bielefeld, Germany

**Keywords:** Habitat, Haemosporidian parasites, Vectors, Ornithophilic simuliidae, Survival, Raptors

## Abstract

Blood parasites of the genus *Leucocytozoon* commonly infect many bird species worldwide and are particularly prevalent in birds of prey. As a vector-borne parasitic disease, the infection occurrence overlaps with that of the dominant vectors: blackflies (Diptera, Simuliidae). These blood-sucking insects are dependent on habitats with flowing freshwaters for the development of their larval stages. We investigated the correlation between the proximity to flowing waters and *Leucocytozoon* infection probability in common buzzard (*Buteo buteo*) broods, as well as the occurrence of adult blackflies directly at the nests. In addition, we investigated the survival of captured simuliids in relation to host infection intensity. In total in 2019, we examined 112 different nests, including 297 common buzzard nestlings, with a *Leucocytozoon* prevalence of 56.6% among the nestlings and of 80.3% at brood level. We found no significant association of *Leucocytozoon* infection probability with nestling age, the distance to the nearest stream and the sum of the length of streams within a radius of 200 and 1000 m around each nest. The number of blackflies caught around the nest showed a tentative correlation with the probability of *Leucocyozoon* infection of the nestlings. Among the subsample of 218 blackfly individuals that survived day one after capture, survival averaged 6.2 days. Our results suggest that *Leucocytozoon* transmission is complex and requires consideration of many factors, related to habitat and vector prevalence, especially given their temporal variation.

## Highlights

• Blood parasite transmission is complex and depends on environmental conditions and vector behaviour.
• In wild common buzzard nestlings, *Leucocytozoon* prevalence was 56.6% of individuals and 80.3% of broods.
• No significant association of *Leucocytozoon* prevalence and distance to the nearest stream and sum of the length of streams within a radius of 200 and 1000 m around each nest were found.
• The number of blackflies caught around the nest tended to explain the *Leucocytozoon* infection probability of nestlings.
• Survival of blackflies in captivity averaged 6.2 days, but did not correlate with infection intensity of the bird hosts.

## Graphical abstract

**Figure:**
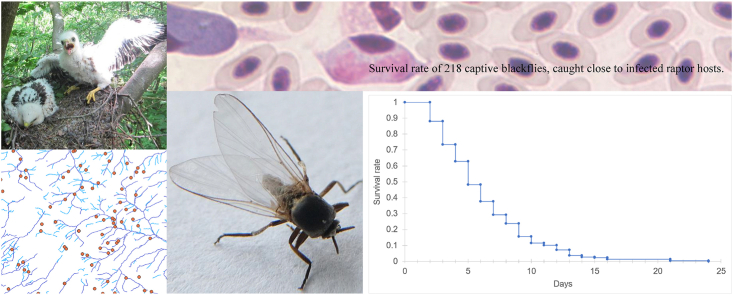

## 1 Introduction

Leucocytozoonosis is a widespread vector-borne infection of birds caused by members of the haemosporidian genus *Leucocytozoon,* occurring globally in numerous bird species (Bensch et al., 2009). To understand the mechanisms behind its epidemiology, it is necessary to consider geographical influences which play a role within the host-vector relationship and support the transmission of parasites (Valkiūnas and Iezhova, 2023). The understanding of avian haemosporidian parasites has greatly benefitted from research in the middle of the 20th century in search of experimental models for human malaria (Davey, 1946; Perkins, 2014). However, *Leucocytozoon* has proven to be the least suitable in this respect. Therefore it remains understudied and poorly understood with few established examples suitable for in-depth explorations. *Leucocytozoon* infections are particularly prevalent in raptors (Krone et al., 2008), which predisposes this group of birds as models for the study of parasite-host interactions.

While research on bird haemosporidian parasites has intensified in recent decades, the detailed understanding of vector biology and local transmission patterns, causing variation in infection rates, is still scarce (Žiegytė and Valkiūnas, 2014). It is known that large river areas are repeatedly affected by mass occurrence of blackflies among other dipteran vectors, which not only pose a risk of infection for humans and mammals with diverse diseases (Dalmat, 1955), but also transmit *Leucocytozoon* spp. among different bird species (Bukaciński and Bukacińska, 2000; Jones et al., 2015). Most research on blackflies has focused primarily on tropical regions and mammalophilic vectors (Adler et al., 2010), while the interrelationships of smaller streams and vector preferences from temperate zones still need detailed investigation. Probably the most famous parasitic disease transmitted by blackflies is onchocerciasis, not least called "river blindness" because of the connection with infection and occurrence of the blackfly vectors close to rivers (Brattig et al., 2021). Therefore, the occurrence as well as the overlap of the breeding habitats of host and vector are probable decisive factors for infection probabilities (Chakarov et al., 2020).

The family Simuliidae contains more than 2000 known species and can be found in a great diversity of habitat types almost everywhere in the world, excluding Antarctica and dry deserts (Adler and McCreadie, 2019; Crosskey, 1990). Notably, the key factor for their life cycle is a dependence and specialization on lotic habitats with freshwater streams and rivers, for the development of their larvae (Adler and McCreadie, 2019). Blackflies are biting Diptera and females of most species require a blood meal from a vertebrate host before oviposition (Crosskey, 1990). These almost exclusively diurnal arthropods are assumed to use olfactory and certain visual cues in their host-seeking strategies, but the process is poorly understood in detail (Adler and Marquardt, 2005). During the blood meal of blackflies, pathogens from infected hosts, like viruses, nematodes and protozoa, can be ingested and through subsequent feeding can be transferred to the next host, potentially leading to serious diseases in humans, domestic and wild animals (Adler and McCreadie, 2019). Avian *Leucocytozoon* spp. are transmitted by blackflies between bird hosts and belong to the group of vector-transmitted protozoa beside the related *Plasmodium* and *Haemoproteus* as well as other unicellular protists like *Trypanosoma* (Adler and Marquardt, 2005). While severe pathogenicity of leucocytozoonosis has been recorded in the poultry industry (Atkinson and van Riper III, 1991), the co-evolution of *Leucocytozoon* and their avian hosts mostly leads to different outcomes under natural conditions (Ashford et al., 1990; Wiegmann et al., 2021). In wild birds, *Leucocytozoon* infections often appear to have little or no effect on health, condition and survival in many studied bird populations (Ashford et al., 1991; Rinaud et al., 2022; Townsend et al., 2018; Wiegmann et al., 2021, Ottensmann et al. in prep.). However, less is known about the effects on the vectors from ingesting *Leucocytozoon*, as well as studies on the general life expectancy of individual blackflies (Davies, 1953). Studying arthropod behaviour still remains challenging and is therefore probably the least studied part of the parasite life cycle (Santiago-Alarcon et al., 2012). The complex development cycle of *Leucocytozoon* includes gut wall penetration within blackflies, which can be expected to lead to increased vector mortality, as known from microfilaria ingestion and other haemosporidians, especially given the large size of *Leucocytozoon* cells compared to other blood parasites (Bukauskaitė et al., 2016; Steele et al., 1992; Takaoka, 2015; Valkiūnas et al., 2014). Potential shortening of life expectancy would be important not only in terms of blackfly biology, but also for *Leucocytozoon* epidemiology and evolution, as vector longevity determines how many reproductive and feeding bouts individual vectors can perform and thus potentially how often they can transmit pathogens (Fischer and Chakarov, 2024; Levin and Parker, 2014).

The aim of this study was to gain insights into the relationships between host, vector and habitat, based on the prevalence patterns in the most common raptor species in Central Europe - the common buzzard, *Buteo buteo*, and its most common blood parasite *Leucocytozoon toddi*. Transmission by blackflies (Diptera: Simuliidae) in these forest raptors is very effective during the nestling period, leading to high infection rates in most years (Chakarov et al., 2008; Wiegmann et al., 2021). The specific habitat and location of the host nest appears to be particularly important for vector-borne infections, and watercourse-rich habitats are typically associated with an increased occurrence of blackflies (Adler et al., 2010). We investigated *Leucocytozoon* infection probabilities of common buzzard broods in relation to the density of watercourses in their nest proximity and the abundance of adult simuliid vectors captured directly at the nests. In addition, the survival of wild-caught blackflies was investigated in captivity to gain insights into the longevity of ornithophilic blackflies and its potential dependence on parasite abundance and infection intensity in the locally available hosts.

## 2 Materials and methods

### 2.1 Study site and geographical measurements

**Fig. 1:**
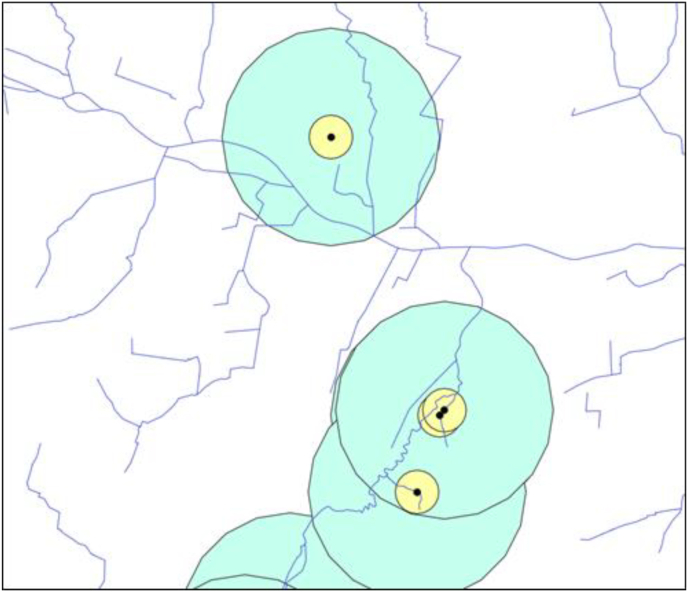
Exemplary graphical excerpt from the QGIS measurement of the sum of the length of streams in a radius of 200 m around the nest (Stream_sum_200m). Scale 1:32714, blue lines: streams, black dots: common buzzard nest, yellow circle: radius of 200 m around a nest, green circle: radius of 1000 m around a nest.

Nests of common buzzards were monitored and nestlings investigated as part of a long-term study of more than two decades in Eastern Westphalia, Germany (Chakarov et al., 2008, 2017). The breeding habitat is described in detail by Krüger (2004) and consists of numerous small woodlots and fields, intersected by many small and medium-sized streams. All nests considered in this study were localized in the federal state North Rhine-Westphalia. Flowing waters within this breeding habitat were recorded by the State Office for Nature, Environment and Consumer Protection North Rhine-Westphalia (LANUV) and provided in an official water stationing map with a positional accuracy up to 3 m (Nordrhein-Westfalen, 2019). The geographical positions of the nests were recorded with Garmin GPS devices and landscape feature analyses were performed with QGIS (Team, 2022). For each nest, the distance to the nearest stream (Dist_to_stream) (Nordrhein-Westfalen, 2019) was calculated in meters. Also, the sum of the length of streams within a radius of 200 and 1000 m around each nest (Stream_sum_200m and Stream_sum_1000m) was measured (Fig. 1).

### 2.2 Sample collection and preparation

All samples were collected within the long-term study of the common buzzard population during the breeding season of spring and summer 2019. Examinations were permitted by the ethics commission of the Animal Care and Use Committee of the German North Rhine-Westphalia State Office for Nature, Environment and Consumer Protection under reference number 84-02.04.2017.A147. For sampling, nestlings were collected from the nests, lowered to the ground and returned to the nest after an examination time of approximately 30 min per brood. Weight, tarsus and wing length were measured for each individual. Nestlings age varied between two and seven weeks within the sampling period (see Wiegmann et al., 2021) for detailed description of the measurement procedure). From each nestling, a blood sample was collected from the ulnar vein by means of a 0.3 mm (30G) x 8 mm needle and two blood smears were prepared and swiftly air-dried for subsequent laboratory investigation. Within 12 h after preparation, these were fixed in absolute ethanol, and subsequently stained with 10 × diluted Giemsa stock solution. All smears were microscopically examined at 400 × magnification (Axioskope, Zeiss, Oberkochen, Germany) and approx. 10.000 erythrocytes per nestling were scanned for haemosporidian parasites. The intensity of infection was categorized as not infected (0 parasites), low level infection intensity (1–10 parasites per 10.000 erythrocytes), medium level infection intensity (>10–100 parasites per 10.000 erythrocytes) and high level infection intensity (>100 parasites per 10.000 erythrocytes).

### 2.3 Blackfly investigation

While the birds were examined on the ground, blackflies (Simuliidae) were caught using a scoop net in the immediate vicinity of the empty nests at a height of 10–30 m in the canopy (Chakarov et al., 2020). Immediately after catching, blackflies were visually examined for a previous blood meal and transferred separately into empty 2 ml tubes, which were covered with a two-layer fly net (1,4 x 1,4 mm mesh size). Tubes with blackflies were stored at room temperature (approx. 20 °C) and an average humidity of approx. 70%. For feeding, each tube was equipped with an approximately 5 × 5 mm piece of sponge, which was soaked with 5% glucose solution, thus lower than used in most dipteran laboratory colonies (e.g. Cupp et al., 1993, Bukauskaitė et al., 2016). Glucose solution was provided ad libitum to the blackflies by refilling the sponge via syringe with a needle when necessary. Every 24 h, blackflies were recorded as alive or dead. Individuals that did not survive the first control on the following day after capture, were recorded as having died on day 0, meaning they survived less than 24 h in captivity, potentially due to capture-induced damage.

### 2.4 Data analysis

Statistical data exploration and analyses were conducted using R 4.0.2 (R Core Team, 2020). The potential correlation of *Leucocytozoon* infection probability with the nest's distance to the nearest stream and the sum of streams within a 200 m and 1000 m radius was investigated among 297 buzzard nestlings, using a generalized linear mixed model (GLMM) with binomial error structure. Due to the fact that an influence of age on infection probability was already shown in a previous study (Wiegmann et al., 2021), standardized age was included as well, while the nest ID was added as a random factor. Age was standardized to a mean of 0 and standard deviation of 1 with the R function “scale”, indicating how much a corresponding nestlings is younger or older than the mean of the sample. The values Dist_to_stream, Stream_sum_200m and Stream_sum_1000m were z-transformed for further comparison in all applied models.

Furthermore, to analyse *Leucocytozoon* infection probability in correlation to the number of blackflies captured per nest (min = 1), a subset of 38 nests where flies were caught with 104 buzzard nestlings was used in another binominal GLMM. Again, standardized age, Stream_sum_200m and Stream_sum_1000m were included as covariates and nest ID was set as a random factor. Finally, the survival probability of blackflies was also assessed by a separate Poisson GLMM with a subset of 218 blackflies, excluding the individuals which died within the first day to account for capture damage. The factors Dist_to_stream, Stream_sum_200m, Stream_sum_1000m and the average brood infection intensity (∅_Parasitemia) were included, in addition to the random factor nest ID. Full models were compared to null models including only the random factor via likelihood ratio tests (R function “anova”).

## 3 Results

### 3.1 *Leucocytozoon* infection and stream habitats

In total, 297 common buzzard nestlings from 112 different broods were investigated. *Leucocytozoon* infections in nestlings were detected in 80.3% of the broods. The general infection probability among the 297 buzzards was 56.6%. The infection intensity was categorized as low in 15.5%, medium in 26.8 % and high in 57.7% of cases. An the nest level, 28.9% of broods contained nestlings with a low average infection intensity (26/90), 40.0% of nests contained nestlings with a medium average infection intensity (36/90), and 31.1% of nests contained nestlings with a high average infection intensity (28/90). The average age of nestlings at the time of examination was 26 days. Thus, on average they had already spent more than half of their nestling period (mean age_standardized 0.59). The distances between the investigated nests to the nearest watercourse varied between 0.2 and 651 m. Within a radius of 200 m around the nests, there was a mean of 373 m of flowing water, within a radius of 1000 m a mean of 6298 m of cumulative streams. Of the three habitat parameters studied, neither distance to the nearest stream, nor the cumulative length of streams within 200 or 1000 m around the nest showed a significant correlation with *Leucozytozoon* infection probability (Table 1).

**Table 1:** Results of the binomial GLMM testing the effect of a) standardized age^a^ and Stream_sum_200m^b^ and b) standardized age^a^, Stream_sum_1000m^b^ and Dist_stream^c^ on the probability of *Leucocytozoon* infection among 297 common buzzard nestlings. The models did not differ significantly from a null model containing only the random factor “nest ID” (model a: χ^2^ = 3.95, Df = 2, *P* = 0.139, model b: χ^2^ = 3.51, Df = 3, *P* = 0.320).

	Estimate	Std. Error	z	*P*
a)				
Intercept	−0.91	0.82	−1.10	0.271
Age_stand	2.085	1.36	1.53	0.127
Stream_sum_200m	−0.178	0.16	−1.11	0.267
b)				
Intercept	−1.00	0.83	−1.20	0.229
Age_stand	2.23	1.37	1.62	0.104
Stream_sum_1000m	0.13	0.17	0.75	0.456
Dist_stream	−0.03	0.18	−0.15	0.884

a For standardization of nestlings' age, each value was divided by the average nestling time of buzzards, so the measure corresponds to the proportion of nestling time spent in the nest at time of sampling.

b Sum of length of flowing waters within a radius of 200 and 1000 m around the common buzzard nest.

c Distance to the nearest stream for each nest.

### 3.2 Stream habitat and blackflies

Blackflies were collected with a scoop net in a subset of 38 nests which included 104 buzzard nestlings. Between one and 24 blackflies were caught per nest with a mean of 6.8 blackflies. No significant correlation was observed between the infection probability and the standardized age nor with stream length (Table 2). There was a tentative correlation between infection probability and the number of blackflies caught around the nest (estimate = 0.05, *P*-value = 0.08).

**Table 2:** Results of a binominal generalized linear mixed model testing the effect of cumulative stream length in a 1000 m radius around the nest, standardized age of common buzzard nestlings and the number of caught blackflies at the nest on the *Leucocytozoon* infection probability among 104 common buzzard nestlings from 38 nests. The full model was not significantly different from a null model containing only the random factor “nest ID” (χ^2^ = 6.73, Df = 3, *P* = 0.081).

	Estimate	Std. Error	z	*P*
Intercept	−1.93	1.28	−1.51	0.131
Sstream_sum_1000m^a^	3.09	2.17	1.43	0.153
Age_stand^b^	0.30	0.22	1.35	0.177
No. of blackflies	0.05	0.03	1.75	0.080

a Sum of length of flowing waters within a radius of 1000 m around the common buzzard nest.

b For standardization of nestlings' age, each value was divided by the average nestling time of buzzards, so the measure corresponds to the proportion of nestling time spent in the nest at time of sampling.

### 3.3 Blackfly survival in captivity

**Fig. 2:**
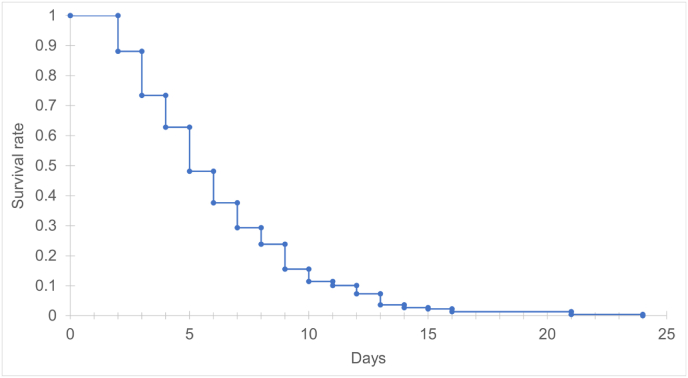
Kaplan-Meier curve for survival rate of 218 captive blackflies that survived at least one day after catch.

In total, 256 blackflies were caught at 38 different nests. Three individuals were apparently engorged with a blood meal. A total of 218 blackflies survived the first day after capture. From this subsample, more than half of the blackflies died within six days after capture (mean survival of 6.2 days/min 2/max 24). The mean survival of engorged blackflies was 4.6 days (min 1, max 8). Details on the survival period of blackflies is presented in Fig. 2. No significant relationship was detected between the lifespan of blackflies and the considered habitat parameters minimum distance to nearest stream, cumulative length of streams within 200 or 1000 m around the nest and the mean infection intensity per nest (Supplementary Table A).

## 4 Discussion

Against expectation, our study showed no correlation between *Leucocytozoon* infection probability of common buzzard nestlings and the distance of the nest to the nearest stream, or different indices of watercourse abundance around the nests. However, the results imply a possible association between the number of blackflies caught per nest with the likelihood of *Leucocytozoon* infection of nestlings. Furthermore, we determined a survival time of up to 24 days in captivity for the simuliids caught at common buzzard nests in the upper canopy.

The *Leucocytozoon* infection probability within the studied habitat of central Europe was high, with more than 80% of common buzzard broods containing at least one infected nestling. This suggests that most of the remaining nestlings within these nests will become infected before fledging and is in line with the general assumption that *Leucocytozoon* infections are widespread in wild birds and particularly so in slowly developing, open-nestling raptors (Atkinson et al., 2008). In the past years, haemosporidian prevalence has been studied in numerous bird species and shown to present high variability in many regions (Bensch et al., 2009). In our study area, both vertebrate hosts and vectors seem to be well adapted to the investigated habitat, which is characterized by fields and woodlands crossed by mainly small streams (less than 0.5 m width). More than half of the studied common buzzard nestlings were infected at an average age of 26 days. This is consistent with our previous study, where transmission already appeared to be very effective in the early life of nestlings and a high prevalence in common buzzard nestlings and other European raptors was found (Wiegmann et al., 2021). However, the exact host-finding mechanisms of the vectors have not been sufficiently investigated so far (Sutcliffe, 2010). Individual characteristics of each host specimen may be important for host preference of black flies (Crosskey, 1990). In addition, infection probabilities appear to depend not only on the bird host species, but also on the breeding location and overlap with vector occurrence, as reported by several authors examining the risk of infection (Bennett, 1960; Gonzalez et al., 2014; Gutiérrez-López et al., 2015; Tella et al., 1999). In a study of vulture broods for example, *Leucocytozoon* prevalence was lower in nests located on cliffs compared to tree nests, which indicates that blackfly vectors may prefer to seek hosts around trees rather than in wide open spaces to avoid the risk of desiccation (Chakarov and Blanco, 2021).

In the present study, we found no significant correlations of *Leucocytozoon* infection probability with the nest proximity to streams, nor to the cumulative stream length in the nest's surroundings. This suggests that more factors need to be taken into account to gain sufficient understanding of the transmission dynamics and host-parasite relationship. The lack of correlation between the studied habitat parameters and infection probability may be due to the complex life cycle of black flies, being strictly dependent on flowing water for their reproductive cycle. The lack of an appreciable correlation between distance to streams and infection probability might be due to the actual high density of small streams in the area which particularly in years with high precipitation might lead to complete saturation of all suitable habitats with vectors. A longitudinal study spanning several years and weather conditions would be needed to support this hypothesis.

Blackfly species differ in preference for water parameters for their larval development (Crosskey, 1990) and different species breed from large rivers to slow flowing streams (Adler and McCreadie, 1997). Therefore, the water conditions for the larvae are crucial for the abundance of single suitable vector species (Olkeba et al., 2022) and these parameters should be included in further studies connecting vector presence and infection probability. On the other hand, transmission patterns can also be influenced by the flight range of adult female blackflies, which can reach up to several kilometers dependent on wind and weather conditions (Crosskey, 1990; Martínez-de la Puente et al., 2009). Transmission and spread of *Leucocytozoon* can also be strongly influenced by blackfly species and their individual characteristics and habitat adaptions (Santiago-Alarcon et al., 2012; Valkiunas, 2005). Environmental factors are key for species richness, whereby among blackflies, habitat generalists and specialist species exist (Ya'cob et al., 2016). Although we did not differentiate the caught blackflies to the species level, a certain diversity of blackfly species is present around raptor nests in this area (Chakarov et al., 2020). Based on a previous study in this high canopy zone, blackflies belonging almost exclusively to the subgenera *Nevermannia* and *Eusimulium* circulate around raptor nests (Chakarov et al., 2020). In contrast to our results, some previous studies could link haemosporidian prevalence and even parasitemia with proximity to water bodies (Fecchio et al., 2021; Ferraguti et al., 2018; Krama et al., 2015; Lachish et al., 2013; Mendenhall et al., 2013; Padilla et al., 2017; Sehgal et al., 2005). However, these have examined mostly *Plasmodium* and *Haemoproteus* parasites, which in contrast to *Leucocytozoon* spp. are not transmitted by blackflies and therefore potentially have different environmental drivers due to vector breeding sites and dynamics. In general, the prevalence of haemosporidian parasites is an ephemeral epidemiological parameter that has both within- and between-season variations, including the peak timing of infections and following occurrence of parasite suppression by host immunity (Santiago-Alarcon et al., 2019). In this study, there was a weak connection between the probability of infection and the number of blackflies caught around the nests. However, the sample size of our dataset may be restrictive and we recommend further investigations on larger data sets. We could not account for blackflies, which may have been present around the nest, but were unreachable through our method of capture through active scoop-netting. After taking a blood meal, blackflies typically depart from the hosts and rest hidden around the nest before their next meal and oviposition (Crosskey, 1990). This is reflected by the fraction of engorged blackflies which we caught. Additionally, in future studies the daytime during examination and its influence on blackfly occurrence at the nests should be considered. Daytime can play a major role in blackfly activity. Different studies present peaks in the morning and afternoon, or midday, but this is strongly depending on the species and environment, as illumination in the tree canopy can be strongly buffered (Crosskey, 1990; Fallis, 1964; Sitarz et al., 2022).

The longevity of vectors likely plays an important role in transmission dynamics, lineage mixing and thus on host-parasite coevolution (Fallis, 1964). We found a maximum life span of simuliids of up to 24 days after capture, comparable with previous studies (Basáñez et al., 1996; Millest et al., 1992; Stanfield, 2003). The average life expectancy commonly ranges between two or three weeks (Crosskey, 1990) and is thus slightly higher than recorded in this study. This can have diverse reasons, e.g. 30%, a greater than elsewhere proportion of blackflies found around the nests appear to be bearers of *Leuocytozoon,* but this may lower their longevity (Chakarov et al., 2020). A peak of mortality in the first day after capture can be explained with injuries and impairments from capture along with heat and transport stress, so we conservatively excluded all blackflies that died within 24 h after capture. In addition, the natural food sources such as nectar and honeydew usually provide higher concentrations of glucose and other sugars than the solution provided in our study with only 5 % glucose (Stanfield, 2003). While feeding on nectar sources belongs to the normal behaviour even of haematophagous species, supply of glucose solution can prolong the survival but also lead to premature death, if flies get trapped by the sticky solution (Stanfield, 2003). In general, a wide variety of laboratory conditions can influence the survival time of vectors (Edman and Simmons, 1985). In addition, some studies have estimated the lifespan of blackflies based on mark recapture methods in the wild, which suggests partly longer lifespan than captivity studies (Bennett and Fallis, 1971; Dalmat, 1952). Therefore, the average life span of 6.2 days is probably an underestimate, compared to natural conditions and demonstrates the lack of established methods for keeping ornithophilic blackflies in laboratory conditions, much less in colony and thus the necessity for establishment of such methods. Additionally, we do not know how long the blackflies had been alive before capture and whether they had already undergone a blood meal and a reproductive cycle or even several.

Despite expectations, the infection intensity within the common buzzard broods did not appear to be correlated with the longevity of blackflies in captivity. In general, infected blood meals have been shown to negatively affect lifespan of dipteran vectors in other studies (Bukauskaitė et al., 2016; Davies, 1953; Valkiūnas and Iezhova, 2004) and the infection intensity of the vertebrate blood meal source seems to be decisive (Takaoka, 2015). Desser and Yang (1973), for example, determined increased mortality within 24 h after a blood meal on heavily infected birds, in comparison to low mortality after lightly *Leucocytozoon* infected blood meals. The average longevity of the three engorged blackflies in this study was slightly lower than the population average, but the effect appears weak due to low sample size. Unfortunately, after they died we did not test whether the collected blackflies were infected with *Leucocytozoon*. The quantification of parasite stages and morphology in the individual vectors and potential dose-specific effects on vector health remains as an important task to be addressed by future studies (Fischer and Chakarov, 2024).

## 5 Conclusions

In summary, in a raptor population with very high infection probability, distance to and density of streams as breeding sites for blackfly vectors did not predict *Leucocytozoon* infection probability within one breeding season. Ornithophilic blackflies have substantial variability in blood meal cycle, longevity, flight range and habitat niche which may complicate the role of this community in terms of simple ecological predictors as well as of their interactions with both avian hosts and blood parasite lineages. Our study indicates that the host seeking preferences of blackflies need further investigation. We could not find an association of blackfly abundance or lifespan with vertebrate host *Leucocytozoon* infection probabilityor intensity so far. A key factor dictating much of *Leucocytozoon* transmission likely depends on the interaction of current, possibly transient environmental conditions and vector behaviour. Therefore, we suggest to consider longer timespans and larger sample sizes in further analyses to reveal epidemiological patterns in wild animal populations as well as to continue searching for suitable conditions for studying simuliids in captivity.

## Funding

This work was supported by the German Research Foundation, DFG, as part of the SFB TRR 212 (NC3) – Project numbers 316099922 and 396780709; DFG project number 398434413; and DFG project number 433069365. The funding bodies played no role in the design of the study and collection, analysis, interpretation of data, and in writing the manuscript.

## Conflict of interest

The authors have no conflict of interest to declare.

## CRediT authorship contribution statement

**Anja Wiegmann:** Writing – original draft, Methodology, Investigation, Formal analysis, Conceptualization. **Andrea Springer:** Writing – original draft, Formal analysis. **Meinolf Ottensmann:** Visualization, Validation, Formal analysis, Data curation, Conceptualization. **Tony Rinaud:** Writing – review & editing, Visualization, Formal analysis, Data curation. **Oliver Krüger:** Writing – review & editing, Validation, Supervision, Conceptualization. **Christina Strube:** Writing – review & editing, Validation, Supervision, Conceptualization. **Nayden Chakarov:** Writing – original draft, Methodology, Conceptualization.

## Declaration of competing interest

None.
